# Interindividual Variation in Chronic Inflammation in Northern Lao People's Democratic Republic

**DOI:** 10.1002/ajpa.70333

**Published:** 2026-08-04

**Authors:** Zhuren Zhang, Mihoko Kibe, Yuki Mizuno, Kazumi Natsuhara, Satoko Kosaka, Hiroaki Masuoka, Kazuhiro Hirayama, Nouhak Inthavong, Sengchanh Kounnavong, Shinsuke Tomita, Masahiro Umezaki

**Affiliations:** ^1^ Department of Human Ecology, School of International Health, Graduate School of Medicine The University of Tokyo Tokyo Japan; ^2^ Faculty of Nursing Toho University Tokyo Japan; ^3^ RIKEN Center for Integrative Medical Sciences Yokohama Japan; ^4^ Graduate School of Agricultural and Life Sciences The University of Tokyo Tokyo Japan; ^5^ Ministry of Health Lao Tropical and Public Health Institute Vientiane Lao PDR; ^6^ Graduate School of Environmental Studies Nagoya University Nagoya Japan

**Keywords:** chronic inflammation, CRP, dietary transition, wild food

## Abstract

**Objectives:**

In many rural regions of developing countries, increasing market access is reshaping local food systems. Traditional diets rooted in local agriculture are being replaced by market‐driven alternatives. It is suspected that this dietary transition will contribute to rising risks of non‐communicable diseases through increased chronic inflammation. This study, conducted in northern Lao People's Democratic Republic, investigated the association between dietary transition and concentrations of C‐reactive protein (CRP) as a biomarker of chronic inflammation in three villages with varying levels of market exposure.

**Methods:**

Data were collected from 380 adults in three villages with different degrees of market integration. Questionnaires recorded individual characteristics and weekly intake frequency of 28 food groups. Dried blood spots were analyzed for CRP using enzyme‐linked immunosorbent assay. Dietary patterns were identified using principal component analysis, and scores were compared among villages. Associations between dietary patterns and CRP were examined using multivariable logistic regression. To account for seasonality, analyses were conducted within same‐season subgroups: rainy (Na Lae, Na Savang) and dry (Na Savang, Nam Nyon).

**Results:**

CRP concentrations were higher in villages with a higher degree of market integration. A dietary pattern was identified, characterized by high consumption of foraged wild plants and a low intake of market‐related foods. This pattern was negatively associated with elevated CRP in the dry season subsample.

**Conclusions:**

Higher CRP concentrations were found in residents of villages with a higher degree of market integration. This trend may partly be explained by a dietary transition from a foraging‐based to a market‐dependent diet.

## 1 Introduction

Even after the advent of agriculture approximately 10,000 years ago, humans continued to gather wild plants and hunt wild animals for subsistence (Somnasang and Moreno‐Black 2000; Bharucha and Pretty 2010; Borelli et al. 2020). As agriculture commercialized, reliance on wild plants and animals gradually declined.

Over the past two centuries, industrialization and globalization have further accelerated these changes. Diets have increasingly incorporated refined grains, sugars, and processed foods, while the diversity of plant and animal sources has narrowed. Unlike earlier generations who relied on a wide array of locally sourced foods, modern humans consume a more monotonous selection shaped by market availability (Carrera‐Bastos et al. 2011).

Ethnographic and nutritional studies have shown that the diets of many subsistence‐farming societies (those with limited exposure to market economies) still feature wild plants and animals to varying degrees (Somnasang and Moreno‐Black 2000; Bharucha and Pretty 2010). These foods can contribute polyunsaturated fats, dietary fiber, and other nutrients, while wild edible plants contain a range of bioactive compounds, most notably phytochemicals (Grivetti and Ogle 2000; Simopoulos 2004; Pinela et al. 2017).

These organic compounds include polyphenols, carotenoids, and terpenes that serve as natural plant defense agents against herbivores, pests, and pathogens (Bennett and Wallsgrove 1994). When consumed by humans, some such compounds modulate oxidative stress and inflammatory pathways both in vitro and in human studies, particularly in the context of traditional herbal medicine (Yeshi et al. 2022; Zhu et al. 2018; Zhang et al. 2015). Modernization has been associated with increasing replacement of wild plant foods by processed, and even ultra‐processed, foods, and intake of potentially beneficial wild compounds has markedly declined (Clemente‐Suárez et al. 2023; Coletro et al. 2023).

In contrast, modern diets share common features such as a high intake of refined grains, added sugars, and saturated fats (Rodríguez‐Monforte et al. 2015; Berg et al. 2008; Shimazu et al. 2007). The transition from traditional to market‐based diets has been associated with elevated levels of inflammatory biomarkers across diverse populations undergoing dietary modernization (Khayyatzadeh et al. 2018; López‐García et al. 2004). Chronic low‐grade inflammation is a risk factor for non‐communicable diseases (NCDs) including metabolic syndrome, type 2 diabetes, cardiovascular disease, and hypertension (Carrera‐Bastos et al. 2011; McDade 2012; O'Keefe and Cordain 2004).

C‐reactive protein (CRP) is a dynamic but non‐specific marker of systemic inflammation. Its level rises rapidly in response to infection or tissue injury but, in the absence of overt acute illness, CRP is commonly interpreted as reflecting background low‐grade inflammation (Pearson et al. 2003). For instance, traditional communities in West Africa and Melanesia have demonstrated lower serum CRP concentrations than European cohorts (Eriksson et al. 2013; Carrera‐Bastos et al. 2020). However, cross‐population comparisons are complicated by genetic variability in CRP expression (Ridker et al. 2008) and uncontrolled socio‐environmental factors such as physical activity.

We therefore hypothesize that the increase in chronic inflammation observed in modern populations is partly attributable to the shift away from traditional dietary patterns and the reduced consumption of phytochemicals.

To evaluate this hypothesis, we conducted field research in the mountainous rural regions of northern Lao People's Democratic Republic (Lao PDR). These areas are home to diverse ethnic minority groups whose traditional subsistence practices, including hunting, gathering, shifting cultivation of upland rice, and wet rice paddy farming, have gradually been replaced by cash cropping. The expansion of road networks and improved logistics have facilitated the emergence of a market economy in what was once a geographically isolated region.

This transformation has resulted in marked inter‐community variation in market integration. Communities deep in the mountains with limited road access continue to rely on subsistence activities, while those near commercial centers are more engaged in secondary and tertiary economic sectors. Villages situated between these extremes typically practice a mix of cash cropping and paddy cultivation. For this study, we selected three villages that represented this gradient of market integration. In this context, market integration is not a linear process that affects diet alone; rather, it is a broader transformation associated with changes in physical activity and exposure to infectious and other environmental conditions. Based on our hypothesis, we focused primarily on the dietary pathway. We assessed dietary patterns, physical activity levels, and each participant's degree of market exposure. Using dried blood spot (DBS) samples, we examined the relationship between these variables and concentrations of CRP, a biomarker of chronic inflammation.

## 2 Materials and Methods

### 2.1 Study Population

The study was conducted in three villages in Oudomxay Province, northern Lao PDR: Na Lae, Na Savang, and Nam Nyon. Na Lae, located in the provincial capital, is the most economically integrated of the three. Residents primarily obtain food and daily necessities from local markets and stores, and many are engaged in retail and cash cropping activities. Na Savang, situated approximately 2 h by car from Na Lae, is a moderately developed village with flat terrain. Wet rice cultivation in paddy fields is the primary livelihood, and local stores provide access to food and other essentials. The village maintains convenient market access via car or motorbike. Nam Nyon, by contrast, is the most remote and least market‐integrated village. Nestled deep in the mountains, it becomes largely inaccessible by car during the rainy season due to poor road conditions. Residents rely heavily on subsistence farming, particularly shifting cultivation of upland rice, and regularly engage in hunting and gathering. Wild boar, small game, and a variety of wild plants from secondary forests are consumed year‐round (Kibe et al. 2022). Surveys were conducted during two distinct seasons: in August 2018 (rainy season) in Na Lae and Na Savang, and in March 2019 (dry season) in Na Savang and Nam Nyon (Mizuno et al. 2022).

### 2.2 Recruitment, Ethics, and Participant Feedback

Adults from the three villages who reported no fever or diarrhea symptoms were invited to participate in the study. We asked leaders to recruit participants in each village. Nam Nyon is an isolated village with a small boundary. The leader used loudspeakers to recruit villagers aged ≥ 18 years in March 2019 (*n* = 146), and the participation rate was approximately 78% (114/146). Na Savang and Na Lae are larger villages with populations of approximately 1000. In these two villages, recruitment was also conducted through loudspeakers by village leaders. However, because it was unclear how many villagers heard the announcements, participation rates could not be calculated. Individuals who agreed to participate received further explanations at designated survey locations and provided written informed consent prior to data collection. Participation was voluntary, and participants were informed that they could decline to commence the study or withdraw at any time without penalty. The study was approved by the research ethics committee of the Graduate School of Medicine, University of Tokyo (approval no. 12033‐(7)), and the National Ethics Committee for Health Research (NECHR), Ministry of Health, Lao PDR (approval numbers: 2018.22.MP, 2022.26, and 2024.61). Study findings were communicated to the participating communities via local feedback sessions and workshops involving Lao health officials and other local stakeholders, thereby ensuring that the results were shared and discussed directly with the communities involved.

The initial participant counts were as follows: 86 (Na Lae, Rainy), 161 (Na Savang, Rainy), 152 (Na Savang, Dry), and 114 (Nam Nyon, Dry). Among the participants from Na Savang, 133 individuals took part in both the rainy‐ and dry‐season surveys.

### 2.3 Demographic and Dietary Assessment

Demographic data were collected through in‐person interviews, including sex, age, smoking status (yes/no), and ownership of six household items (cell phone, motorcycle, car, truck, television, and refrigerator) to assess economic status. These items were selected based on field observations in the study area. At our study sites, when households began to earn more cash, they usually bought mobile phones first, then motorcycles and refrigerators, and later, larger assets such as trucks. Cars were much less common, being owned by only a small number of better‐off households. As ownership tended to increase step‐by‐step in this manner, each item was scored as 1 if owned and 0 if not, yielding a wealth index ranging from 0 to 6. At the village level, the mean or median wealth index was used to represent the overall level of market integration and the relative affluence of each community. At the individual level, the wealth index served as a proxy for a household's purchasing power and socioeconomic status.

Dietary data were gathered using a locally adapted questionnaire that covered 28 food groups to allow assessment of individual dietary variation within the study area, including wild plant, wild animal, cultivated, and market or processed foods (e.g., wild vegetables and fruits, bush meat, cultivated vegetables, and canned fish; see Table 1). These food groups were not intended to represent the village‐specific food cultures, being rather foods that were widely consumed across the region. The consumption frequencies varied by the context, such as the distance to markets or the frequency of foraging. Participants reported the number of days each food group was consumed over the previous 7 days (0 to 7 days). Only consumption frequency, not portion size or total caloric intake, was recorded.

**TABLE 1:** Food grouping in the food frequency questionnaire.

Food groups	Food items
Rice	Rice (dry/wet)
Corn	Corn
Tuber crops	Tuber crops (e.g., Taro, sweet potato, cassava etc.)
Meats	Meats (water buffalo, cattle, pig, goat, dog etc.)
Poultry/Eggs	Poultry/Eggs
Cultivated vegetables	Cultivated leafy vegetables (e.g., Chinese cabbage)
Cultivated fruits	Cultivated fruits
Herbs	Herbs
Spices	Spices (e.g., Chili pepper, galangal, ginger, Chinese pepper)
Fish	Fish
Aquatic animals	Other aquatic animals (frog, crab, shrimp etc.)
River weed	River weed and Algae
Bitter bamboo	Bitter bamboo shoot
Wild fruits	Wild fruits
Wild vegetables	Wild vegetables
Bush meats	Bush meats
Insects	Insects or larvae
Organs of animals	Organs of animals
Contents of intestine	Contents of intestine from fish, buffalo or cattle
Blood of animals	Blood of animals (Duck, pig, water buffalo, wild animals etc.)
Organs of fish	Organs of fish
Coffee/Tea	Coffee/tea
Noodles	Noodles
Processed meat	Sausages, other processed meat
Canned fish	Canned fish
Sweets	Sweets and snacks
Oil/Fat	Oil/Fat
Alcohols	Alcohols

Physical activity was assessed using a modified version of the Global Physical Activity Questionnaire (GPAQ) (Armstrong and Bull 2006). Participants indicated the number of hours and days they had engaged in moderate and vigorous physical activity during the previous week. These responses were used to estimate weekly hours of moderate to vigorous physical activity (MVPA).

### 2.4 Anthropometric Assessment and DBS Sampling

Anthropometric measurements, including height and weight, were obtained using a mobile stadiometer (Holtain Ltd., Crosswell, UK) and a digital scale (Tanita, Tokyo, Japan). DBS samples were collected via fingerprick using sterile lancets (Becton, Dickinson and Company, US) and applied to protein saver cards (Whatman, UK). In each village, sample collection was conducted over 3 days. In the field, DBS samples were dried at the sampling site under ambient temperature. After drying, the DBS cards were sealed in plastic bags with desiccant and placed in portable cooling containers and transferred to a household freezer on the same day (after the morning and afternoon collection sessions had been completed). The samples were then stored in the household freezer for approximately 6 days. After the survey had been completed, all samples were transported in cooling containers to the Lao Tropical and Public Health Institute, Vientiane, Lao PDR, and stored at −80°C. They were then transferred, frozen, to the University of Tokyo laboratory and stored at −80°C until analysis.

### 2.5 Measurement of CRP

CRP concentrations were analyzed using a modified ELISA protocol based on Brindle et al. (2010). The assay utilizes mouse monoclonal antibodies to measure CRP from DBS samples. Calibrators and controls were prepared following the method established by McDade et al. (2004).

DBS samples were eluted from filter paper using a 1/8‐in. punch, equivalent to approximately 1.525 μL of serum (Mei et al. 2001), in assay buffer. Jansen et al. (2015) reported that serum samples stored at −80°C exhibited superior stability compared to those stored at −20°C. Accordingly, our DBS samples were stored at −80°C for 27 months prior to analysis. All samples were run in duplicate. Samples with an intra‐assay coefficient of variation (CV) exceeding 20% were reassayed. For CRP concentrations below the lower limit of detection, values were imputed as half the detection limit. Samples exceeding the upper detection limit were reassayed using a higher dilution factor. To minimize inter‐assay variability, capture and signal antibodies from a single production lot were used throughout the study.

The average inter‐assay CV for controls was 14.8%, with values of 14.4% for low control, 11.0% for medium control, and 19.0% for high control (*N* = 17 plates × 3 controls per plate). Intra‐assay variation, calculated from duplicate measurements of 513 samples, yielded an average CV of 6.04%. Detailed information on detection limits and assay variability is provided in Table S1.

### 2.6 Statistical Methods

The U.S. Centers for Disease Control and Prevention and the American Heart Association classify CRP levels as follows: < 1 mg/L indicates low risk for cardiovascular disease, 1–3 mg/L indicates average risk, and > 3 mg/L indicates high risk. These cutoff points are derived from approximate tertiles of CRP concentrations based on data from over 15 populations and more than 40,000 participants (Pearson et al. 2003). Additionally, CRP concentrations > 10 mg/L are typically attributed to acute infection. However, these cutoffs were primarily established using data from populations living in industrialized environments. In high‐pathogen rural settings, moderate infections and intestinal parasites are common, rendering it difficult to determine whether a moderately elevated CRP level reflects background chronic inflammation or a transient inflammatory response (Wander et al. 2012; Peto et al. 2016; Binnie et al. 2020). Given our focus on chronic inflammation among participants without significant underlying disease, we applied a more stringent threshold, excluding samples with CRP concentrations above 5.0 mg/L. Samples whose CRP concentrations remained above the assay detection range even after reassay using a higher dilution factor, indicating values above the quantifiable range, also exceeded the 5.0 mg/L threshold and were therefore excluded from the analytical models. As previously noted, data were collected during the rainy season of 2018 and the dry season of 2019. Samples from both seasons were obtained only in Na Savang village. After excluding samples above 5.0 mg/L, the final analytic sample sizes were 85 (Na Lae, Rainy), 157 (Na Savang, Rainy), 142 (Na Savang, Dry), and 106 (Nam Nyon, Dry).

Seasonal variations in dietary patterns and physical activity were considered in the analysis. Na Lae, being more market‐integrated, was expected to show less seasonal fluctuation in diet compared to the more subsistence‐based villages of Na Savang and Nam Nyon, where wild‐resource availability varies seasonally. Similarly, physical activity levels in Na Savang and Nam Nyon were anticipated to differ between seasons due to agricultural labor demands. Therefore, data were analyzed separately by season: the rainy season group included Na Lae and Na Savang, while the dry season group included Na Savang and Nam Nyon. Comparisons of basic characteristics and multivariable regression analyses were conducted within each seasonal group.

Principal component analysis (PCA) was used to derive dietary patterns from the food frequency data. Food groups consumed by fewer than 20% of participants (e.g., insects, herbs, processed meat) were excluded, as was rice, which was consumed daily by over 99% of participants. Prior to analysis, all frequency variables were standardized (*z*‐transformed to a mean of 0 and a standard deviation of 1). PCA was conducted on the standardized frequency variables without rotation. The number of components used in subsequent analyses was determined based on the proportion of explained variance, visual inspection of the scree plot, the decrease in explained variance between successive components, and the interpretability of the component loadings. The resulting principal component scores, referred to as dietary‐pattern scores, served as proxies for dietary trends in subsequent analyses. Spearman's correlation coefficients between food group consumption frequencies and component scores were also calculated to describe the associations between individual food groups and the derived components.

To compare general characteristics such as sex, smoking status, and wealth‐index scores across villages, chi‐square tests were used. Differences in BMI and dietary‐pattern scores were assessed using *t*‐tests. Wilcoxon signed‐rank tests were applied to compare age, wealth index scores, MVPA hours, and CRP concentrations between villages.

Logistic regression analysis was performed with elevated CRP concentrations as the outcome variable. Dietary‐pattern scores and physical activity (MVPA hours) were the main explanatory variables, while wealth index scores, BMI values, age, sex, and smoking status were included as covariates. Interaction terms between dietary‐pattern scores and BMI values were also included. As CRP concentrations typically exhibit a right‐skewed distribution, previous studies have commonly used natural log transformation when treating the CRP level as a continuous variable. However, this did not work for our dataset because a substantial proportion of the measurements was below the assay detection limit (Figure S1). Thus, CRP values were dichotomized using the clinical cutoff (i.e., CRP ≥ 1.0 mg/L). This separated individuals in a low‐risk category from those in average‐to‐high relative cardiovascular risk categories as defined by Pearson et al. (2003). Interaction terms between dietary‐pattern scores and BMI values were added because it was anticipated that the potential anti‐inflammatory effects of wild‐plant foods might not be detectable in individuals with a low BMI value, who would be expected to already be at low risk of chronic inflammation (Visser et al. 1999). Before constructing the interaction terms, BMI values were mean‐centered to facilitate the interpretation of interaction effects. Age, sex, and smoking status are associated with CRP levels (Tracy et al. 1997; Tang et al. 2018; Chai et al. 2016). The home village was included as a covariate to control for site‐specific environmental differences, such as variation in market access. Because approximately two participants came from each household on average, we also tested a household random effect. However, its estimated variance was essentially zero, suggesting little additional between‐household variation after other covariates were included. We therefore used clustered robust standard errors at the household level to account for residual non‐independence (Cameron and Miller 2015). To further assess general trends across CRP categories beyond the binary classification, an ordered logistic regression analysis was performed using established CRP categories: low (< 1.0 mg/L), intermediate (1.0 to < 3.0 mg/L), and high (3.0 to ≤ 5.0 mg/L, given the exclusion of CRP values > 5.0 mg/L) (Pearson et al. 2003). This model included the same main effect covariates as the binary logistic regression models, without interaction terms. Clustered robust standard errors were calculated at the household level.

To examine the interrelationships among variables identified in the main analyses further, an exploratory piecewise structural equation model (pSEM) was constructed. In this model (Lefcheck 2016), age, sex, village, and wealth index scores served as upstream socio‐demographic and economic variables; the *forager* dietary‐pattern score (see Section 3.1), MVPA hours, and smoking status served as behavioral variables; BMI scores served as an intermediate physiological indicator; and an elevated CRP level (≥ 1.0 mg/L) served as the final health outcome. This model was used to examine market integration, viewed as a complex system of interrelated socio‐economic, behavioral, and physiological factors linked to chronic inflammation.

All analyses were performed using R software (version 4.2.1; R Core Team, 2022).

## 3 Results

### 3.1 Identified Dietary Patterns

PCA was applied to the reported consumption frequency of 24 food groups. Two principal components were retained based on a combination of criteria, including the proportion of explained variance (> 10%); visual inspection of the scree plot (Figure S2); a marked drop in explained variance after the second component (from 0.11 to 0.06, followed by only small and gradual declines); and the interpretability of the resulting dietary patterns. The loadings for each food group on the first and second components are presented in Table 2, along with Spearman's correlation coefficients between food group consumption frequencies and the respective component scores.

**TABLE 2:** Assessment of factor‐loading matrix of dietary principal components and Spearman correlation coefficients between dietary pattern scores and consumption frequency of each food group.

	Forager		Organ	
	Factor‐loading	Spearman correlation coefficient	Factor‐loading	Spearman correlation coefficient
Wild vegetables	0.27	0.46***	−0.17	0.27***
Bitter bamboo	0.26	0.44***	−0.14	0.24***
River weed	0.24	0.4***	−0.16	0.27***
Aquatic animals	0.17	0.29***	0.28	0.45***
Bush meats	0.14	0.24***	0.29	0.48***
Alcohols	0.10	0.17***	−0.08	0.13**
Contents of intestine	0.10	0.16***	0.44	0.72***
Tuber crops	0.08	0.13**	−0.15	0.25***
Blood of animals	0.07	0.12*	−0.14	0.24***
Organs of fish	0.05	0.09*	0.44	0.72***
Organs of animals	−0.07	−0.11*	0.36	0.59***
Canned fish	−0.07	−0.11*	0.21	0.34***
Cultivated vegetables	−0.07	−0.12**	0.00	0
Wild fruits	−0.09	−0.15**	−0.18	0.3***
Spices	−0.15	−0.26***	0.04	−0.07*
Oil/Fat	−0.17	−0.29***	−0.07	0.11*
Fish	−0.19	−0.32***	−0.16	0.27***
Poultry/Eggs	−0.23	−0.38***	−0.09	0.15**
Corn	−0.24	−0.4***	−0.16	0.26***
Coffee/Tea	−0.25	−0.41***	−0.05	0.08
Sweets	−0.26	−0.43***	−0.16	0.27***
Noodles	−0.27	−0.45***	−0.13	0.21***
Cultivated fruits	−0.38	−0.63***	−0.12	0.2***
Meats	−0.40	−0.68***	−0.06	0.1*
Proportion of variance	0.12		0.11	

* *p* < 0.05.

** *p* < 0.01.

*** *p* < 0.001.

Based on the loading patterns, the first principal component was interpreted as a *forager* dietary pattern, characterized by positive associations with foods obtained through foraging activities (such as wild vegetables, bitter bamboo, and river weed) and negative associations with market‐related foods (including meats, cultivated fruits, noodles, sweets, coffee/tea, and poultry/eggs). The second component was labeled the *organ* dietary pattern, defined by high consumption of animal and fish organs, bush meats, aquatic animals, and canned fish. The *forager* and *organ* dietary patterns explained 12% and 11% of the total variance, respectively.

### 3.2 Comparison of General Characteristics and DBS CRP Between Villages

Table 3 summarizes participants' general characteristics, anthropometric data, DBS CRP concentrations, and dietary‐pattern scores by village and season. After excluding samples with CRP concentrations above 5.0 mg/L, the final analytic sample sizes were 85 (Na Lae, Rainy), 157 (Na Savang, Rainy), 142 (Na Savang, Dry), and 106 (Nam Nyon, Dry).

**TABLE 3:** Basic characteristics of participants based on villages and seasons.

	Rainy season			Dry season		
	Na Lae	Na Savang	*p* ^a^	Na Savang	Nam Nyon	*p* ^a^
*N* ^b^	85	157		142	106	
Sex = male (%)	25 (29.4)	61 (38.9)	0.185	54 (38.0)	49 (46.2)	0.244
Age (years) (median [IQR])	44 [34, 52]	41 [32, 48]	0.053	42 [34, 50]	32 [27, 43]	**< 0.001**
Smoking = yes (%)	12 (14.1)	28 (17.8)	0.574	26 (18.3)	42 (39.6)	**< 0.001**
BMI (mean (SD))	24.68 (3.80)	22.49 (3.37)	**< 0.001**	23.06 (3.46)	22.27 (3.32)	0.073
MVPA (hours/week) (median [IQR])	5.3 [0.6, 14.5]	11.0 [4.1, 28.0]	**< 0.001**	11.5 [4.0, 22.5]	49.0 [18.3, 58.3]	**< 0.001**
CRP (mg/L) (median [IQR])	0.69 [0.32, 1.27]	0.41 [0.15, 0.86]	**0.01**	0.57 [0.24, 1.44]	0.46 [0.14, 0.86]	0.056
Possessions						
Phone = yes (%)	81 (95.3)	155 (98.7)	0.228	137 (96.5)	57 (53.8)	**< 0.001**
Motorbike = yes (%)	80 (94.1)	149 (94.9)	1	137 (96.5)	55 (51.9)	**< 0.001**
Car = yes (%)	18 (21.2)	36 (22.9)	0.88	29 (20.4)	0 (0.0)	**< 0.001**
Truck = yes (%)	2 (2.4)	6 (3.8)	0.815	5 (3.5)	1 (0.9)	0.374
Television = yes (%)	84 (98.8)	151 (96.2)	0.441	136 (95.8)	73 (68.9)	**< 0.001**
Refrigerator = yes (%)	78 (91.8)	88 (56.1)	**< 0.001**	84 (59.2)	4 (3.8)	**< 0.001**
Wealth index (median [IQR])^c^	4 [4, 4]	4 [3, 4]	**0.001**	4 [3, 4]	2 [1, 3]	**< 0.001**
Dietary pattern scores^d^						
*Forager* (mean (SD))	−2.12 (1.79)	−0.35 (0.97)	**< 0.001**	0.36 (1.00)	1.73 (0.97)	**< 0.001**
*Organ* (mean (SD))	−0.81 (1.36)	0.04 (1.85)	**< 0.001**	0.48 (1.71)	−0.07 (1.13)	**0.004**

*Note:* Bold values indicate statistical significance (*p* < 0.05).

Abbreviations: BMI, body mass index; CRP, high‐sensitivity C‐reactive protein; IQR, interquartile range; MVPA, moderate‐to‐vigorous physical activity; SD, standard deviation.

^a^ The Chi‐square test was applied for factors including gender, smoking status, and poverty ownership. The *t*‐test was applied for BMI and dietary pattern scores. The wilcoxon signed rank test was applied for age, wealth index, MVPA, and CRP.

^b^ The sample size and percentage of samples lower than the detection limit (0.104 mg/L) across groups are: Rainy Na Lae (*N* = 12, 14.12%), Rainy Na Savang (*N* = 37, 23.57%), Dry Na Savang (*N* = 14, 9.86%), and Dry Nam Nyon (*N* = 24, 22.64%).

^c^ Wealth index was calculated as the sum of the numbers of possessed properties.

^d^ Dietary pattern scores represent individual principal component (PCA) scores, calculated as the weighted sum of standardized food group consumption frequencies multiplied by their respective PCA loadings.

**FIGURE 1:**
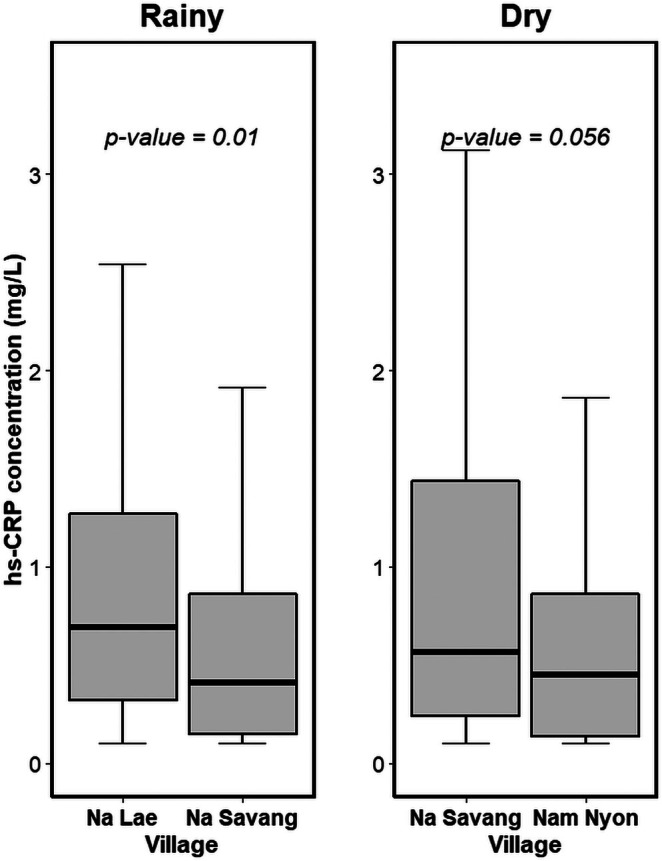
Comparison of CRP between villages. Data are presented as medians with interquartile ranges (IQR). Statistical comparisons between villages were conducted using the Wilcoxon rank‐sum test. Sample sizes were: Na Lae Rainy (*N* = 85), Na Savang Rainy (*N* = 157), Na Savang Dry (*N* = 142), and Nam Nyon Dry (*N* = 106). Villages represent different levels of market integration, with Na Lae being the most modernized, Na Savang intermediate, and Nam Nyon the least modernized. CRP, C‐reactive protein.

In the rainy season sample, Na Lae exhibited higher BMI and wealth‐index values compared to Na Savang. Residents of Na Lae reported lower weekly MVPA hours, as well as lower scores for both the *forager* and *organ* dietary patterns. In the dry‐season sample, participants in Na Savang were older and had higher BMI values, wealth‐index values, and *organ* dietary‐pattern scores than those in Nam Nyon. Smoking prevalence, weekly MVPA hours, and *forager* dietary‐pattern scores were higher in Nam Nyon than in Na Savang. Figure 1 compares DBS CRP concentrations across villages. During the rainy season, the concentrations were significantly higher in Na Lae than in Na Savang. In the dry season, they were higher in Na Savang than in Nam Nyon, although the difference was only marginally significant (*p* = 0.056).

### 3.3 Multivariable Logistic Regression

Multivariable logistic regression analyses were conducted separately for each season, with elevated CRP (≥ 1.0 mg/L) as the outcome variable. As shown in Table 4 (Model 1), no significant associations were observed between dietary‐pattern scores and CRP values in the rainy season sample (Na Lae, Na Savang). In contrast, within the dry season sample (Na Savang, Nam Nyon), the *forager* dietary‐pattern score was significantly and negatively associated with elevated CRP values.

**TABLE 4:** Multivariate logistic regression analyses with CRP^a^ as dependent variables.

Variables	Model 1^b^				Model 2^b^			
	Rainy season (Na Lae, Na Savang)		Dry season (Na Savang, Nam Nyon)		Rainy season (Na Lae, Na Savang)		Dry season (Na Savang, Nam Nyon)	
	Odds ratio (95% CI)^d^	*p*	Odds ratio (95% CI)^d^	*p*	Odds ratio (95% CI)^d^	*p*	Odds ratio (95% CI)^d^	*p*
Sex (ref. = female) Male	0.60 (0.23–1.60)	0.31	0.73 (0.31–1.72)	0.468	0.60 (0.23–1.58)	0.303	0.77 (0.32–1.83)	0.554
Age (years)	0.99 (0.96–1.03)	0.791	1.02 (0.99–1.05)	0.126	1.00 (0.96–1.03)	0.794	1.02 (0.99–1.05)	0.144
BMI	1.31 (1.17–1.47)	**< 0.001**	1.16 (1.06–1.28)	**< 0.001**	—	—	—	—
BMI (mean‐centered)^c^	—	—	—	—	1.31 (1.14–1.50)	**< 0.001**	1.30 (1.13–1.50)	**< 0.001**
MVPA (hours/week)	1.00 (0.99–1.02)	0.653	0.99 (0.98–1.01)	0.506	1.00 (0.99–1.02)	0.655	0.99 (0.98–1.01)	0.377
Smoking (ref. = No) Yes	1.23 (0.38–3.97)	0.723	0.87 (0.31–2.38)	0.78	1.24 (0.39–3.94)	0.714	0.82 (0.30–2.28)	0.708
Wealth index	0.96 (0.68–1.35)	0.81	0.73 (0.52–1.02)	0.065	0.96 (0.67–1.36)	0.807	0.71 (0.50–1.00)	**0.047**
Dietary pattern scores								
*Forager*	0.86 (0.67–1.09)	0.205	0.75 (0.56–0.99)	**0.042**	0.86 (0.66–1.11)	0.25	0.74 (0.55–1.00)	**0.049**
*Organ*	0.99 (0.83–1.18)	0.929	0.99 (0.82–1.19)	0.897	1.01 (0.83–1.23)	0.896	1.02 (0.84–1.23)	0.872
BMI × *Forager*	—	—	—	—	1.00 (0.93–1.06)	0.939	0.89 (0.82–0.98)	**0.013**
BMI × *Organ*					1.00 (0.94–1.06)	0.871	1.02 (0.97–1.07)	0.48
Village (ref. = Na Savang) Na Lae	0.81 (0.33–1.99)	0.65	—	—	1.23 (0.50–3.01)	0.652	—	—
Village (ref. = Na Savang) Nam Nyon	—	—	0.73 (0.34–1.59)	0.425	—	—	0.71 (0.33–1.56)	0.394

*Note:* Village was included as a fixed effect to control for site‐specific environmental variance. Bold values indicate statistical significance (*p* < 0.05).

Abbreviations: BMI, body mass index; CI, confidence interval; CRP, C‐reactive protein; MVPA, moderate‐to‐vigorous physical activity.

^a^ Elevated CRP concentration was defined as > 1.0 mg/L.

^b^ Model 1 includes main effects only; Model 2 additionally includes an interaction term between BMI (mean‐centered) and the *Forager* dietary pattern.

^c^ BMI was mean‐centered in interaction models to improve the interpretability of main effects and reduce multicollinearity.

^d^ Standard errors were clustered at the household level (Household ID), and corresponding 95% confidence intervals and *p*‐values were calculated accordingly.

To further examine whether these associations varied across levels of adiposity, interaction terms between BMI and dietary pattern scores were included in the secondary models in Table 4 (Model 2). No significant interaction effects were observed in the rainy season sample. In the dry season sample, whereas no significant interaction was observed between BMI and the *organ* dietary‐pattern score, a statistically significant interaction was observed between BMI and the *forager* dietary‐pattern score (OR = 0.89, 95% CI = 0.82–0.98). It was interesting that the OR of the interaction term between BMI and the *forager* dietary‐pattern score was lower than 1.0, which indicates that *forager* dietary pattern is most strongly inversely associated with CRP > 1 mg/L at the highest BMIs and most weakly associated with CRP > 1 mg/L at the lowest BMIs. Each 1 kg/m^2^ increase in BMI multiplied this odds ratio by 0.89. Additionally, the wealth index was inversely associated with elevated CRP in this model.

The results of the ordered logistic regression analyses were broadly consistent with the binary logistic regression models (Table S2). The *forager* dietary‐pattern score was not significantly associated with CRP categories in the rainy season sample (OR = 0.88, 95% CI: 0.70–1.10, *p* = 0.260) but was inversely associated with higher CRP categories in the dry season sample (OR = 0.73, 95% CI: 0.55–0.95, *p* = 0.022). BMI was positively associated with higher CRP categories in both seasons.

### 3.4 pSEM

**FIGURE 2:**
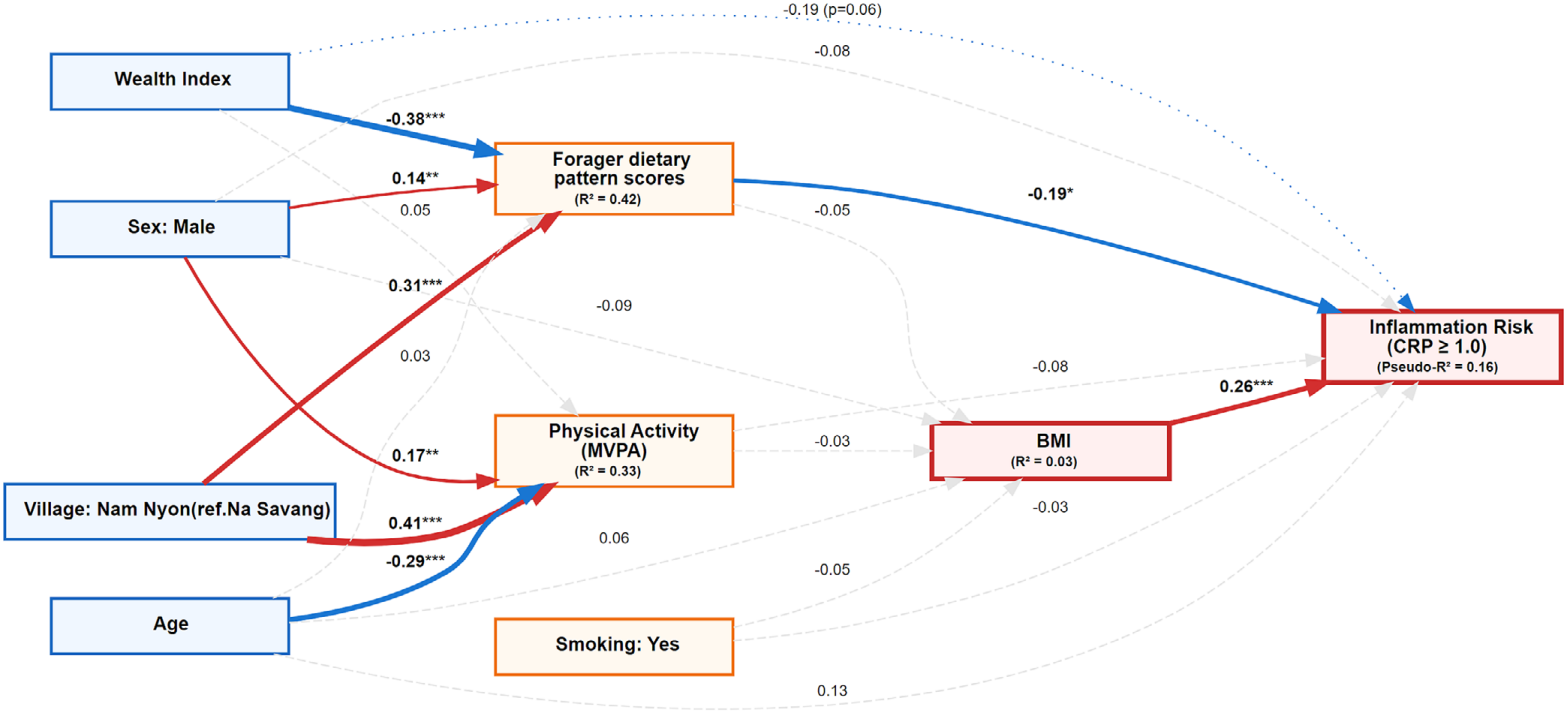
Results of the exploratory piecewise structural equation model (pSEM) for the dry season subgroup (Na Savang and Nam Nyon). Rectangles indicate variables included in the model, grouped by role: Blue boxes represent upstream socio‐demographic and economic variables (wealth index, sex, village, and age), orange boxes represent behavioral variables (*forager* dietary‐pattern score, moderate‐to‐vigorous physical activity [MVPA], and smoking), and red boxes represent physiological and health outcomes (BMI and elevated CRP). Solid arrows indicate hypothesized paths retained in the fitted model; blue arrows indicate negative associations and red arrows indicate positive associations. Gray dashed arrows indicate tested paths that were not statistically significant. Values shown next to arrows are standardized path coefficients. Asterisks indicate statistical significance (*p < 0.05, **p < 0.01, ***p < 0.001); the dotted blue path from wealth index to elevated CRP indicates a marginal association (*p* = 0.06). *R* ^2^ values are shown within endogenous variables: Values for the *forager* dietary‐pattern score, MVPA, and BMI are from linear component models, whereas the value for elevated CRP is Nagelkerke's pseudo‐*R* ^2^ from the logistic component model. Overall model fit was acceptable (test of directed separation: Fisher's *C* statistic = 15.62, degrees of freedom = 12, *p* = 0.209; Chi‐square = 7.16, degrees of freedom = 6, *p* = 0.306).

The results of the pSEM are shown in Figure 2, and the detailed model coefficients are listed in Table S3. The overall fit of the model was acceptable (Fisher's *C* = 15.62, *p* = 0.209; *χ* ^2^ = 7.16, *p* = 0.306), implying that the hypothesized structure was broadly consistent with the data. In the model, higher wealth‐index scores were associated with lower *forager* dietary‐pattern scores, whereas residence in Nam Nyon and male sex were associated with higher *forager* dietary‐pattern scores. MVPA hours were higher in Nam Nyon and among men, but decreased with age. Elevated CRP levels were directly associated with lower *forager* dietary‐pattern scores and higher BMI values, whereas a direct association with wealth‐index values was only marginal, and MVPA hours were not significantly related to CRP levels. BMI values were only weakly explained by the upstream variables included in the model.

## 4 Discussion

### 4.1 Village‐Level Variation Across the Market Integration Gradient

Throughout the three villages, increasing market integration was associated with consistent differences in wealth, physical activity, diet, and BMI values. Wealth‐index values were higher in the more market‐integrated villages (Na Lae > Na Savang > Nam Nyon), reflecting increased access to cash income and more purchase of market goods. In contrast, MVPA hours were lower in the more market‐integrated settings (Na Lae < Na Savang < Nam Nyon), implying a shift away from physically demanding activities such as farming and foraging toward more sedentary routines. The dietary patterns followed the same trend. *Forager* dietary‐pattern scores were lower in the more market‐integrated villages (Na Lae < Na Savang < Nam Nyon), indicating reduced reliance on wild foods and greater consumption of market‐based foods. Mean BMI values were also higher in the more market‐integrated villages, consistent with the observed changes in diet and physical activity.

### 4.2 Dietary Transition as a Pathway

The observed negative association between *forager* dietary‐pattern scores and CRP levels implies that dietary transition was a key pathway linking market integration to inflammatory status. These findings support the hypothesis that reduced consumption of wild‐sourced foods under market integration may contribute to chronic low‐grade inflammation.

In addition, the interaction between BMI values and the *forager* dietary‐pattern scores suggests that the inflammatory implications of dietary transition may depend partly on adiposity. This implies that dietary change may not exert a uniform effect across all individuals; rather, its association with CRP levels may be more apparent among those with higher BMI values, whose baseline inflammatory status is already elevated. However, BMI does not distinguish fat mass from lean mass. In physically active individuals, especially in less market‐integrated communities, higher BMI may partly reflect greater muscle mass rather than adiposity.

A key aspect of dietary transition under market integration is a reduction in dietary diversity, particularly declining reliance on wild‐sourced foods. Reyes‐García et al. (2019) demonstrated that market accessibility was strongly associated with dietary diversity among contemporary hunter‐gatherers. In the cited study, Tsimane people living in remote areas consumed a wider variety of foods, including more wild‐sourced fruits and vegetables, compared to those residing near markets. The same study also reported a similar pattern among the Baka of Cameroon. Importantly, increased market integration was associated with reduced intake of nutritionally important foods such as fruits, vegetables, and animal products and increased consumption of dietary sugars and fats. Our findings, which show a decreasing trend in wild‐food consumption with greater market integration, are consistent with those observations.

Modern diets are often characterized by features that contrast with region‐specific, subsistence‐based, or minimally processed dietary patterns. Several studies have linked modern dietary patterns to chronic inflammation. For instance, research on the Samoa Islands, where Westernization has driven significant dietary and health transitions, has identified a modern dietary pattern via PCA analysis, marked by high intake of sausages, eggs, and processed foods rich in refined grains (e.g., potato chips, instant noodle soup, pancakes). This pattern has been positively associated with elevated serum triglycerides and increased prevalence of metabolic syndrome (Wang et al. 2017). Similarly, a study in Jiangsu, China, found that a Westernized dietary pattern, characterized by high consumption of pork, poultry, vegetables, fruits, dairy products, and whole grains, was positively associated with metabolic syndrome prevalence (Wang et al. 2021).

While PCA‐defined “Western” or “Modern” dietary patterns vary by region, they often share common features: high intake of meat from mammals, refined grains, processed meats, dairy products, sweets, and desserts (Wang et al. 2017, 2021; Rodríguez‐Monforte et al. 2015; Brennan et al. 2010). In our study, several food groups that loaded negatively on the *forager* dietary pattern, such as sweets, meats, poultry/eggs, noodles, and corn, align with these previously identified modern dietary components.

It is important to note that in the rainy season sample (Na Lae and Na Savang), no significant association was observed between the *forager* dietary pattern and CRP values. One limitation of our dietary assessment is that the questionnaire captured only the number of days each food group was consumed in the previous week, without accounting for portion sizes or total caloric intake. This may have obscured meaningful variation in dietary quantity, particularly among residents of Nam Nyon, who reported substantially higher weekly MVPA (median of 49 h) compared to the other villages (Na Savang Rainy: 11 h; Na Savang Dry: 11.5 h; Na Lae Rainy: 5.3 h). It is plausible that individuals with higher physical activity levels also consumed larger quantities of wild plants and overall calories, differences not reflected in our data and potentially contributing to the seasonal discrepancies in observed associations.

Additionally, no significant association was found between CRP values and the *organ* dietary pattern in either season. In contrast, a questionnaire‐based anti‐inflammatory diet index developed for Nordic populations classified organ meats as pro‐inflammatory and reported a positive association with CRP levels (Kaluza et al. 2018). Our findings did not replicate this trend. A plausible explanation is that the organ meats consumed in these Lao communities differ from those in European contexts, being freshly sourced and unprocessed. Moreover, given that our questionnaire did not capture portion sizes or caloric intake, it is possible that organ meats were consumed in small quantities, limiting their detectable impact on CRP levels.

### 4.3 Impact of Reduced Consumption of Wild Plants

Building on our hypothesis, a reduced intake of phytochemicals from wild resources appears to be a significant contributor to increased chronic inflammation. Numerous in vitro and human studies have demonstrated the anti‐inflammatory properties of phytochemicals derived from traditional herbal medicines (Yeshi et al. 2022; Zhu et al. 2018; Zhang et al. 2015). This trend is compounded by increased consumption of pro‐inflammatory components commonly found in modern dietary patterns.

In our study, wild vegetables, bitter bamboo, and river weed loaded positively on the *forager* dietary pattern. The “wild vegetables” category in our questionnaire included both fully wild species and semi‐cultivated plants growing near fields or in secondary forests. In contrast, river weeds and algae are entirely wild and not cultivated. Table S4 lists the plant species commonly consumed during the dry and rainy seasons in Nam Nyon village, based on our previous fieldwork (Kibe et al. 2022).

Although fewer species of wild plants and river weeds were consumed during the dry season, several notable items, including mustard greens ( *Brassica juncea* ), algae ( *Cladophora glomerata* ; *Spirogyra* species), and bitter bamboo (*Indosasa sinica*), were exclusively consumed during this period, when the Nam Nyon samples were collected. *Brassica juncea* is rich in vitamin A, vitamin C, phenolic compounds, and glucosinolates, and its antioxidant and anti‐inflammatory effects have been well documented in both in vivo and in vitro studies (Tian and Deng 2020). Ethanol extracts of *Brassica juncea* have been shown to reduce levels of tumor necrosis factor‐α (TNF‐α), interleukin (IL)‐1β, and IL‐6 mRNA in mice exposed to inflammatory stimuli (Xian et al. 2018). Algae also possess potent antioxidant and anti‐inflammatory properties due to their abundance of bioactive compounds, including phenolics and sterols. In vivo and in vitro studies have demonstrated anti‐inflammatory effects for *Cladophora glomerata* and *Spirogyra* (Bourebaba et al. 2019; Wang et al. 2020).

In a previous field study conducted in Nam Nyon village, we observed that villagers consumed wild plants at almost every meal, accounting for more than 20% of their total dietary intake by weight (Kibe et al. 2022). Based on our findings and previous research on the antioxidant and anti‐inflammatory properties of these wild plants, it is plausible that regular consumption of wild plants contributes to lower concentrations of CRP. There are more species if we consider wild plants eaten during the rainy season and those consumed for non‐habitual and medicinal purposes. The anti‐inflammatory effects of a long‐term intake of all these wild plants might operate throughout the seasons and years.

Together, these results imply that the elevation in CRP levels corresponding to higher degrees of market integration across the three villages can be attributed to a dietary shift characterized by increased consumption of pro‐inflammatory foods and reduced intake of wild plants abundant in phytochemicals.

### 4.4 Physical Activity

A growing body of research has examined patterns of physical activity in subsistence populations undergoing market integration. Studies on the Tsimane of Bolivia suggested that increasing market exposure did not necessarily reduce overall physical activity, as market participation often remained embedded in labor‐intensive subsistence and wage activities (Gurven et al. 2013). Similarly, research on Shuar children found no clear difference in activity levels between rural and peri‐urban settings, although market food consumption was associated with increased adiposity (Urlacher et al. 2021).

These findings indicate that the effects of market integration on physical activity are not uniform, rather depending on the specific forms of livelihood transition in play. In our study, MVPA hours varied across villages in a pattern consistent with differences in market integration. The transition from subsistence farming and foraging to mixed livelihoods including retail activities was associated with a reduction in MVPA hours. This implies that levels of physical activity are shaped by changing livelihood structures, rather than declining uniformly with market exposure.

However, despite this variation, MVPA hours were not significantly associated with CRP concentrations. This indicates that, in the context of this study, a change in physical activity was not the primary pathway linking market integration to inflammatory outcomes.

Although higher levels of physical activity are often inversely associated with systemic inflammation, the evidence is not entirely consistent. In a multi‐ethnic cohort, Majka et al. (2009) reported that the relationship between physical activity and CRP levels varied by ethnicity and sex, exhibiting inverse or null patterns across various subgroups, and no such relationship was evident in Chinese participants, who also exhibited the lowest CRP levels. Our sample consisted of ethnic minorities from Northern Lao PDR, who exhibit genetic affinities to Chinese populations and a low median CRP level, which may have limited the detectable associations.

### 4.5 Wealth Index and Infectious Disease

In the dry season sample, a notable trend emerged regarding the relationship between wealth index values and CRP concentrations. This finding contrasts with our initial hypothesis that greater economic affluence, associated with market integration, would lead to increased CRP levels due to a shift away from traditional foraging practices toward market‐based food choices. As shown in Table 3, wealth index values increased with market integration, while *forager* dietary‐pattern scores decreased. However, after adjusting for BMI values and dietary‐pattern scores, the regression analysis revealed an inverse association between wealth index values and CRP levels.

One plausible explanation is that, once dietary patterns are accounted for, wealth index values may reflect other factors that influence inflammation, such as improved household infrastructure and sanitation. These non‐dietary components of socioeconomic status may contribute to lower systemic inflammation by reducing exposure to environmental pathogens. This hypothesis is further supported by the distribution of acute inflammatory markers across our study sites. In our sample, the prevalence of individuals with CRP concentrations exceeding 5.0 mg/L—our primary threshold for excluding potential acute inflammation—varied across the subgroups: 1.2% (*n* = 1) in Na Lae (rainy season), 2.5% (*n* = 4) in Na Savang (rainy season), 6.6% (*n* = 10) in Na Savang (dry season), and 7.0% (*n* = 8) in Nam Nyon (dry season). When applying the clinical threshold of CRP > 10.0 mg/L, the relative proportions decreased to 1.2% (*n* = 1), 1.9% (*n* = 3), 3.3% (*n* = 5), and 4.4% (*n* = 5) respectively. Notably, as the degree of market integration increased, the proportion of individuals experiencing acute inflammatory spikes tended to decrease, whereas the overall population mean CRP level tended to rise. This paradox may underscore a transition in the immune profile from pathogen‐driven acute responses to metabolically driven chronic low‐grade inflammation.

However, given the absence of direct diagnostic data on acute or sub‐clinical infections (e.g., pathogen loads or symptomatic records), we cannot definitively detail the precise contribution of infectious diseases to the observed CRP data. For this reason, we adopted a threshold of 5 mg/L, rather than 10 mg/L, to reduce the likelihood that undetected acute or sub‐clinical infections influenced the sample. As wealth may alter both pathogen exposure and lifestyle, this implies that market integration reflects a highly complex set of processes. Thus, the observed association between wealth‐index values and CRP levels may represent only one aspect of several underlying immunological and environmental shifts.

### 4.6 Integration of Behavioral and Biological Pathways

The pSEM indicated that the impact of market integration on subsistence occurred via multiple pathways rather than a single sequential track. Differences in upstream socio‐demographic and economic factors were linked to variations in *forager* dietary‐pattern scores and MVPA hours, indicating that village context and wealth influenced behavioral change via distinct mechanisms. However, these behavioral variables did not consistently predict downstream biological outcomes. Neither *forager* dietary‐pattern scores nor MVPA hours were significantly associated with BMI values, whereas elevated CRP levels were associated with lower *forager* dietary‐pattern scores and higher BMI values. Among the pathways examined, that of diet demonstrated the strongest association with CRP levels. As *forager* dietary‐pattern scores were based on food‐consumption frequency rather than portion size or caloric intake, this result should not be interpreted as evidence of increased energy intake. Rather, it implies that qualitative dietary changes, particularly reduced reliance on wild‐food‐related dietary components, were associated with variation in chronic inflammation status independently of BMI values.

### 4.7 Limitations

This study had several limitations. First, seasonal disparity between the two sampling periods may have influenced the results, as dietary patterns and food availability likely varied not only by village but also by season. Although we analyzed the data in two seasonal subsets rather than pooled models to account for this variation, doing so reduced statistical power due to smaller sample sizes.

Second, the food‐frequency questionnaire did not capture portion sizes or caloric intake, limiting our ability to assess total dietary load or energy‐adjusted associations. While we hypothesized that phytochemicals in wild plants may contribute to reduced inflammation, we were unable to analyze their effects directly. The supporting evidence cited for antioxidant and anti‐inflammatory properties was drawn from previous in vivo and in vitro studies, often conducted in different ecological contexts. Future research should prioritize the extraction and analysis of phytochemical constituents from locally consumed wild‐plant species to elucidate their potential anti‐inflammatory effects within the study population.

Third, although individuals with fever or diarrhea and those with CRP values above 5.0 mg/L were excluded, subclinical or asymptomatic infections may still have influenced the CRP concentrations. In a high‐pathogen setting such as northern Lao PDR, it remains difficult to distinguish fully background low‐grade inflammation from infection‐related inflammation. In addition, as the infectious disease burden may differ across villages, especially between more and less remote communities, these exclusion criteria may also have unequally affected the composition of the analytical sample across the sites. As a result, some observed between‐village differences may partly reflect residual variation or differential exclusion related to infectious disease rather than dietary or market‐integration processes alone.

Fourth, participants were recruited through convenience recruitment rather than random sampling. This approach may have affected participation across communities due to village leadership and individual availability. For example, in villages where leaders had stronger relationships with residents or greater persuasive power, recruitment may have reached a broader range of residents. Residents in more market‐integrated villages may have been unavailable because of wage labor, whereas residents in less market‐integrated villages may have been absent due to subsistence activities. Such possibilities cannot be fully ruled out. However, based on our repeated visits and fieldwork in these communities, we do not expect that this convenience recruitment method introduced substantial bias into our analytical results.

Fifth, BMI was used as a proxy for adiposity, but it does not distinguish fat mass from lean mass. In physically active populations, higher BMI may partly reflect greater muscle mass rather than higher adiposity. This limitation may have affected our interpretation of associations involving BMI.

Finally, the wealth index used in this study captured only one dimension of market integration and may also have reflected broader differences in household environments, living conditions, and exposure contexts across the villages. Because this study was cross‐sectional, the study communities may have differed in their experiences of market integration at the time of data collection. These limitations imply that our findings should be interpreted as evidence for one plausible pathway linking market integration with inflammation, rather than a complete account of all mechanisms involved.

## 5 Conclusion

This study identified inter‐village differences in DBS CRP concentrations among ethnic minorities in rural regions of northern Lao PDR. Residents of villages with greater market integration exhibited higher CRP concentrations, implying a link between economic transition and systemic inflammation. Within the dry‐season sample, the *forager* dietary pattern, characterized by frequent consumption of wild plants and limited intake of market‐based foods, was significantly and negatively associated with CRP concentrations, even after adjusting for age, sex, smoking status, and BMI values. These findings imply that dietary transitions accompanying market integration, particularly reduced reliance on wild‐plant foods and the associated potential decline in phytochemical‐rich dietary resources, may contribute to elevated chronic inflammation.

## Author Contributions

**Mihoko Kibe:** writing – review and editing, investigation. **Zhuren Zhang:** conceptualization, methodology, validation, data curation, formal analysis, writing – original draft, writing – review and editing. **Yuki Mizuno:** investigation, writing – review and editing. **Hiroaki Masuoka:** investigation, writing – review and editing. **Satoko Kosaka:** investigation, writing – review and editing. **Shinsuke Tomita:** conceptualization, data curation, funding acquisition, investigation, project administration, writing – review and editing. **Sengchanh Kounnavong:** project administration, writing – review and editing. **Kazuhiro Hirayama:** investigation, writing – review and editing. **Nouhak Inthavong:** data curation, investigation, writing – review and editing, project administration. **Kazumi Natsuhara:** investigation, writing – review and editing. **Masahiro Umezaki:** conceptualization, funding acquisition, investigation, project administration, supervision, writing – original draft, writing – review and editing.

## Funding

This work was supported by Japan Society for the Promotion of Science, JP17H02233, JP19H03315, JP20K21443, JP21H03684, JP23K17525.

## Ethics Statement

This study was approved by the research ethics committee of the Graduate School of Medicine, University of Tokyo (approval number: 12033‐(7)) and the National Ethics Committee for Health Research (NECHR), Ministry of Health, Lao PDR (approval numbers: 2018.22.MP, 2022.26, and 2024.61).

## Conflicts of Interest

The authors declare no conflicts of interest.

## Supporting information


**Table S1:** Detection limit and inter‐ and intra‐assay CVs (%) of CRP measurement.
**Table S2:** Ordered logistic regression analyses with hs CRP‡ categories as dependent variables.
**Table S3:** Results of piecewise SEM.
**Table S4:** Wild plants regularly consumed in Nam Nyon village.
**Figure S1:** Distribution of CRP by season and transformation scale. (A) Raw CRP concentrations in the dry season; (B) Log‐transformed CRP concentrations in the dry season; (C) Raw CRP concentrations in the rainy season; (D) Log‐transformed CRP concentrations in the rainy season. The dashed red lines in panels (A) and (C) indicate the lower limit of detection (LLOD = 0.104 mg/L). CRP, C‐reactive protein; CRP, C‐reactive protein; LLOD, lower limit of detection.
**Figure S2:** Scree plot and cumulative proportion explained from principal component analysis (PCA) of reported consumption frequencies for 24 food groups. The scree plot displays the eigenvalues for each principal component, while the cumulative proportion line shows the proportion of total variance explained as components are added. Two components with a proportion of variance > 0.10 were retained to define the dietary patterns used in the analysis.

**Data S2:** Supporting Information.

## Data Availability

Because the study villages were very small, individual participants could potentially be identified from a combination of village, sex, and age. Therefore, full individual‐level data supporting the findings of this study cannot be shared publicly. The dataset to be made publicly available includes only village, sex, CRP, and age grouped into 10‐year categories. The publicly available dataset is provided as Supporting Information S2 accompanying this article.
