# Supplementary material for: Interindividual Variation in Chronic Inflammation in Northern Lao People's Democratic Republic
Source: Am J Biol Anthropol. 2026 Aug 4;190(4):e70333. doi: 10.1002/ajpa.70333 (PMC13438680; doi:10.1002/ajpa.70333)
Supplement: Supplementary file 1 [file AJPA-190-e70333-s002.doc]

| Table S1. Detection limit and inter- and intra-assay CVs (%) of CRP measurement | | |  |
| --- | --- | --- | --- |
| Detection limit (mg/L) | inter-assay CV (%)^†^ | intra-assay CV (%)^‡^ |  |
| 0.104 | 14.80 | 6.04 |  |
| ^†^ Average value of inter-assay CV (%) of 3 controls (14.4 = low control, 11.0 = medium control, 19.0 = high control), N = 17 plates × 3 controls per plate | |  |  |
| ^‡^ Average value of intra-assay CV (%), N = 513 | | |  |
|  |  |  |  |

| Table S2. Ordered logistic regression analyses with hs CRP^‡^ categories as dependent variables | | | |  |
| --- | --- | --- | --- | --- |
|  | Rainy season (Na Lae, Na Savang) | | Dry season (Na Savang, Nam Nyon) | |
| Variables | Odds ratio (95% CI)^†^ | *p*-value | Odds ratio (95% CI)^†^ | *p*-value |
| Sex (ref. = female) Male | 0.59 (0.24–1.47) | 0.26 | 0.74 (0.31–1.76) | 0.497 |
| Age (years) | 0.99 (0.96–1.03) | 0.744 | 1.02 (1.00–1.05) | 0.102 |
| BMI | 1.30 (1.17–1.44) | **<0.001** | 1.14 (1.05–1.24) | **0.002** |
| MVPA (hours/week) | 1.01 (0.99–1.03) | 0.381 | 1.00 (0.98–1.02) | 0.882 |
| Smoking (ref. = No) Yes | 1.15 (0.37–3.53) | 0.809 | 0.88 (0.32–2.44) | 0.806 |
| Wealth index | 0.97 (0.70–1.33) | 0.839 | 0.75 (0.56–1.02) | 0.067 |
| Dietary pattern scores |  |  |  |  |
| *Forager* | 0.88 (0.70–1.10) | 0.26 | 0.73 (0.55–0.95) | **0.022** |
| *Organ* | 1.02 (0.86–1.21) | 0.816 | 1.02 (0.85–1.22) | 0.868 |
| Village (ref. = Na Savang) Na Lae | 0.84 (0.35–2.01) | 0.696 | — | — |
| Village (ref. = Na Savang) Nam Nyon | — | — | 0.74 (0.35–1.58) | 0.442 |
| ^†^ Standard errors were clustered at the household level (Household ID), and corresponding 95% confidence intervals are shown. | | | | |
| ^‡^ The dependent variable was ordered hs CRP category: low (< 1.0 mg/L), intermediate (1.0 to < 3.0 mg/L), and high (3.0 to ≤ 5.0 mg/L). In the complete case samples, the rainy season model included 241 participants (low = 180, intermediate = 50, high = 11), and the dry season model included 248 participants (low = 175, intermediate = 54, high = 19). | | | | |
| Village was included as a fixed effect to control for site-specific environmental variance. | | |  |  |
| **Abbreviations:** MVPA, moderate-to-vigorous physical activity; BMI, body mass index; CRP, C-reactive protein; CI, confidence interval. | | | | |

| Table S3. Results of piecewise SEM |  |  |  |  |  |
| --- | --- | --- | --- | --- | --- |
| Response Variable | Predictor | Estimate^†^ | Standard Error | Critical Value | P-Value |
| Forager Diet Score | Wealth Index | -0.3181 | 0.0566 | -5.6189 | **< 0.001** |
| (R2=0.42) | Age | 0.0026 | 0.0053 | 0.4923 | 0.6229 |
|  | Sex (ref. = female) Male | 0.3358 | 0.119 | 2.8205 | **0.0052** |
|  | Village (Nam Nyon) | 0.7521 | 0.1638 | 4.5915 | **< 0.001** |
| MVPA (hours/week) | Wealth Index | 0.8311 | 1.2597 | 0.6598 | 0.51 |
| (R2=0.33) | Age | -0.611 | 0.1174 | -5.2031 | **< 0.001** |
|  | Sex (ref. = female) Male | 8.5721 | 2.6485 | 3.2365 | **0.0014** |
|  | Village (Nam Nyon) | 20.412 | 3.6446 | 5.6007 | **< 0.001** |
| BMI | Forager Diet | -0.1419 | 0.1965 | -0.7221 | 0.4709 |
| (R2=0.03) | MVPA | -0.0047 | 0.0102 | -0.4567 | 0.6483 |
|  | Age | 0.0166 | 0.0202 | 0.8226 | 0.4116 |
|  | Smoking (ref. = No) Yes | -0.3644 | 0.7108 | -0.5127 | 0.6086 |
|  | Sex (ref. = female) Male | -0.6276 | 0.6243 | -1.0053 | 0.3157 |
| Elevated CRP (>1.0mg/L) | Wealth Index | -0.2673 | 0.1401 | -1.9075 | 0.0565 |
| (Pseudo-R2^‡^=0.16) | *Forager* dietary pattern score | -0.3152 | 0.1604 | -1.9645 | **0.0495** |
|  | BMI | 0.1509 | 0.0442 | 3.4189 | **< 0.001** |
|  | Age | 0.0216 | 0.0141 | 1.5319 | 0.1255 |
|  | Sex (ref. = female) Male | -0.3171 | 0.4495 | -0.7056 | 0.4805 |
|  | Smoking (Yes) | -0.1486 | 0.5293 | -0.2807 | 0.7789 |
|  | MVPA | -0.0065 | 0.0074 | -0.8818 | 0.3779 |

Fisher’s C⁵ = 15.62, degrees of freedom = 12, p = 0.209; Chi-square = 7.16, degrees of freedom = 6, p = 0.306

^†^For the categorical response variable Elevated CRP, estimates are reported as log-odds; for all other response variables, estimates are unstandardized coefficients.

^‡^Nagelkerke’s pseudo-R² is reported for the CRP model; adjusted R² is reported for the other component models.
⁵ A non-significant Fisher’s C indicates adequate overall fit of the piecewise structural equation model.

**Abbreviations:** CRP, C-reactive protein; MVPA, moderate-to-vigorous physical activity; BMI, body mass index; SEM, structural equation model.

| Table S4. Wild plants regularly consumed in Nam Nyon village | | |
| --- | --- | --- |
|  | Common names, groups | Species |
| Dry season | Mustard greens | *Brassica juncea* |
|  | Algae | *Cladophora glomerata; Spirogyra sp.* |
| Rainy season | Cucumber leaves and fruits | *Cucumis sativus L.* |
|  | Fern | *Diplazium esculentum* |
|  | Cassava leaf | *Manihot esculenta* |
|  | Solanum | *Solanum sp.* |
|  | Melon | *Cucumis melo* |
| Throughout the year | Bitter bamboo | *Indosasa sinica* |

Fig S1. Distribution of CRP by season and transformation scale. (A) Raw CRP concentrations in the dry season; (B) Log-transformed CRP concentrations in the dry season; (C) Raw CRP concentrations in the rainy season; (D) Log-transformed CRP concentrations in the rainy season. The dashed red lines in panels (A) and (C) indicate the lower limit of detection (LLOD = 0.104 mg/L).

Abbreviations: CRP, C-reactive protein; CRP, C-reactive protein; LLOD, lower limit of detection.

Fig S2. Scree plot and cumulative proportion explained from principal component analysis (PCA) of reported consumption frequencies for 24 food groups. The scree plot displays the eigenvalues for each principal component, while the cumulative proportion line shows the proportion of total variance explained as components are added. Two components with a proportion of variance > 0.10 were retained to define the dietary patterns used in the analysis.
